# Associations of total amount and patterns of sedentary behaviour with type 2 diabetes and the metabolic syndrome: The Maastricht Study

**DOI:** 10.1007/s00125-015-3861-8

**Published:** 2016-02-02

**Authors:** Julianne D. van der Berg, Coen D. A. Stehouwer, Hans Bosma, Jeroen H. P. M. van der Velde, Paul J. B. Willems, Hans H. C. M. Savelberg, Miranda T. Schram, Simone J. S. Sep, Carla J. H. van der Kallen, Ronald M. A. Henry, Pieter C. Dagnelie, Nicolaas C. Schaper, Annemarie Koster

**Affiliations:** Department of Social Medicine, Maastricht University, PO Box 616, 6200 MD Maastricht, the Netherlands; CAPHRI School for Public Health and Primary Care, Maastricht University, Maastricht, the Netherlands; Department of Internal Medicine, Maastricht University Medical Centre+, Maastricht, the Netherlands; CARIM School for Cardiovascular Diseases, Maastricht University, Maastricht, the Netherlands; Department of Human Movement Sciences, Maastricht University, Maastricht, the Netherlands; NUTRIM School for Nutrition and Translational Research in Metabolism, Maastricht University, Maastricht, the Netherlands; Department of Epidemiology, Maastricht University, Maastricht, the Netherlands

**Keywords:** Accelerometry, Diabetes mellitus type 2, Metabolic syndrome, Sedentary bouts, Sedentary breaks, Sedentary lifestyle, Sedentary time

## Abstract

**Aims/hypothesis:**

The study investigated cross-sectional associations of total amount and patterns of sedentary behaviour with glucose metabolism status and the metabolic syndrome.

**Methods:**

We included 2,497 participants (mean age 60.0 ± 8.1 years, 52% men) from The Maastricht Study who were asked to wear an activPAL accelerometer 24 h/day for 8 consecutive days. We calculated the daily amount of sedentary time, daily number of sedentary breaks and prolonged sedentary bouts (≥30 min), and the average duration of the sedentary bouts. To determine glucose metabolism status, participants underwent an oral glucose tolerance test. Associations of sedentary behaviour variables with glucose metabolism status and the metabolic syndrome were examined using multinomial logistic regression analyses.

**Results:**

Overall, 1,395 (55.9%) participants had normal glucose metabolism, 388 (15.5%) had impaired glucose metabolism and 714 (28.6%) had type 2 diabetes. The odds ratio per additional hour of sedentary time was 1.22 (95% CI 1.13, 1.32) for type 2 diabetes and 1.39 (1.27, 1.53) for the metabolic syndrome. No significant or only weak associations were seen for the number of sedentary breaks, number of prolonged sedentary bouts or average bout duration with either glucose metabolism status or the metabolic syndrome.

**Conclusions/interpretation:**

An extra hour of sedentary time was associated with a 22% increased odds for type 2 diabetes and a 39% increased odds for the metabolic syndrome. The pattern in which sedentary time was accumulated was weakly associated with the presence of the metabolic syndrome. These results suggest that sedentary behaviour may play a significant role in the development and prevention of type 2 diabetes, although longitudinal studies are needed to confirm our findings.

## Introduction

Type 2 diabetes mellitus is a chronic disease with high prevalence and incidence worldwide [1] that, next to its classic complications such as cardiovascular disease and retinopathy, can cluster with other chronic diseases such as dementia and chronic obstructive pulmonary disease (COPD). During the last decades several risk factors, including genetic, environmental and lifestyle factors, have been identified as relevant for the development of type 2 diabetes [2, 3], but these cannot fully explain its development. Recent research interest is therefore focusing on the role of newly identified determinants, such as sedentary behaviour.

Sedentary behaviour, defined as any waking behaviour characterised by an energy expenditure ≤ 1.5 metabolic equivalents (METs) while in a sitting or reclining posture, such as watching TV or using the computer [4], can be objectively measured using an accelerometer. A number of accelerometry studies have shown unfavourable associations between total amount of sedentary time and metabolic health outcomes, including waist circumference [5–9], cholesterol and triacylglycerol levels [5, 6, 10–13], markers of insulin resistance [5, 6, 9, 10, 13, 14] and the metabolic syndrome [8, 11, 15–17]. Apart from the total amount of sedentary time, the pattern of sedentary time, i.e. the frequency with which sedentary time is interrupted (sedentary breaks) or the duration of uninterrupted periods of sedentary time (sedentary bouts), seems to be relevant for health outcomes. In a few studies, more sedentary breaks have been associated with better metabolic health [15, 18, 19].

To date, large-scale studies that have objectively measured sedentary behaviour in a population with type 2 diabetes have been scarce [5, 17]. However, given the large amounts of time people spend being sedentary and the high prevalence of type 2 diabetes, such studies are important. These studies can provide more insights into the associations between sedentary behaviour and diabetes, and contribute to the development of strategies to prevent diabetes and its complications and comorbidities. Therefore, we measured the total amount and patterns of sedentary behaviour with an accelerometer in a large sample of adults with type 2 diabetes, impaired glucose metabolism (IGM) or normal glucose metabolism (NGM), who participated in The Maastricht Study. We used the thigh-worn activPAL3 accelerometer, which classifies sedentary behaviour using data on posture, as this has been shown to be an accurate means of assessing sedentary behaviour [20, 21]. The aim of the present study was to examine associations of total amount and patterns of sedentary behaviour with glucose metabolism status and the metabolic syndrome.

## Methods

In this study, we used data from The Maastricht Study, an observational, prospective, population-based cohort study. The rationale and methods have been described previously [22]. In brief, the study focuses on the aetiology, pathophysiology, complications and comorbidities of type 2 diabetes mellitus and is characterised by an extensive phenotyping approach.

Eligible participants were individuals aged between 40 and 75 years and living in the southern part of the Netherlands. Participants were recruited through mass media campaigns and from the municipal registries and the regional Diabetes Patient Registry via mailings. Recruitment was stratified according to known type 2 diabetes status for reasons of efficiency. This study included cross-sectional data from 3,451 participants who completed the baseline survey between November 2010 and September 2013. After excluding participants who did not receive an accelerometer due to logistics (*n* = 673), whose accelerometer measurement failed (*n* = 136) or who had other missing data (*n* = 145), a total of 2,497 participants were included in the present analyses.

The study was approved by the institutional medical ethical committee (NL31329.068.10) and the Minister of Health, Welfare and Sports of the Netherlands, on the basis of the Health Council’s opinion (permit 131088-105234-PG). All participants gave written informed consent.

## Measurements

### Glucose metabolism status and the metabolic syndrome

To determine glucose metabolism status, all participants (except those who used insulin) underwent a standardised 75 g oral glucose tolerance test after an overnight fast, as described elsewhere [22]. Glucose metabolism was defined according to the World Health Organization’s 2006 criteria [23], and participants were categorised as having NGM, impaired fasting glucose (fasting plasma glucose 6.1–6.9 mmol/l and 2 h plasma glucose [after glucose load] < 7.8 mmol/l), impaired glucose tolerance (fasting plasma glucose < 7.0 mmol/l and 2 h plasma glucose [after glucose load] ≥7.0–11.1 mmol/l), or type 2 diabetes mellitus (fasting plasma glucose ≥7.0 mmol/l or 2 h plasma glucose [after glucose load] ≥11.1 mmol/l). Participants on diabetes medication and without type 1 diabetes were also considered to have type 2 diabetes. For this study, we defined having either impaired fasting glucose and/or impaired glucose tolerance as IGM.

To determine the metabolic syndrome, we measured, as described elsewhere [22]: waist circumference, triacylglycerol levels, HDL-cholesterol levels, fasting glucose levels, blood pressure and medication use. The metabolic syndrome was defined according to the Adult Treatment Panel (ATP) III guidelines by the presence of three or more of: (1) waist circumference ≥102 cm for men or ≥88 cm for women; (2) serum triacylglycerol level ≥1.7 mmol/l; (3) HDL-cholesterol level <1.03 mmol/l for men or <1.30 mmol/l for women; (4) fasting glucose level ≥5.6 mmol/l or use of glucose-lowering drug medication (insulin or oral agents); or (5) systolic blood pressure ≥130 mmHg and/or diastolic blood pressure ≥85 mmHg, and/or use of blood-pressure-lowering medication [24].

### Sedentary behaviour variables

Sedentary time was measured using the activPAL3 physical activity monitor (PAL Technologies, Glasgow, UK). The activPAL3 is a small (53 × 35 × 7 mm), lightweight (15 g) triaxial accelerometer that records movement in the vertical, anteroposterior and mediolateral axes, and also determines posture (sitting or lying, standing and stepping) based on acceleration information. The device was attached directly to the skin on the front of the right thigh with transparent 3M Tegaderm tape, after the device had been waterproofed using a nitrile sleeve. Participants were asked to wear the accelerometer for 8 consecutive days, without removing it at any time. To avoid inaccurately identifying non-wear time, participants were asked not to replace the device once removed. Data were uploaded using the activPAL software and processed using customised software written in MATLAB R2013b (MathWorks, Natick, MA, USA). Data from the first day were excluded from the analysis because participants performed physical function tests at the research centre after the device was attached. In addition, data from the final wear day providing ≤14 waking hours of data were excluded from the analysis. Participants were included if they provided at least 1 valid day (>14 h of waking data).

The total amount of sedentary time was based on the sedentary posture (sitting or lying), and calculated as the mean time spent in a sedentary position during waking time per day. The method used to determine waking time has been described elsewhere [25]. In brief, an automated algorithm identified wake and bed times on an individual level on multiple days, i.e. different wake and bed times for each day for each participant. The algorithm is based on the number and duration of sedentary periods to identify bed times, and on the number and duration of active periods (standing or stepping) to identify wake times. The algorithm showed high accuracy in determining waking time compared with self-report, as the intra-class correlation coefficient (ICC) was 0.79 (*p* < 0.001) and the mean difference in waking time between both methods was 0.02 h (1.2 min), with limits of agreement of −1.1 to 1.2 h.

The number of sedentary breaks during waking time was determined as each transition from a sitting or lying position to standing or stepping, and the mean number of breaks per day was calculated. Sedentary time accumulated in a consecutive period ≥30 min was defined as a prolonged sedentary bout, and the mean number of prolonged sedentary bouts during waking time per day was calculated. Average bout duration was calculated by dividing total sedentary time by the total number of sedentary bouts. Minutes with a step frequency **>** 110 steps/min were classified as higher intensity physical activity and this variable was used as a measure for moderate to vigorous physical activity (MVPA) [26].

### Covariates

Covariates that were extracted from the questionnaire included sex, age, level of education, smoking status, alcohol consumption, mobility limitation, health status and diabetes duration. Level of education was categorised as low, medium or high, and smoking status as never, former or current smoker. Alcohol consumption was categorised as non-consumer, low consumer (≤7 alcoholic drinks per week for women and ≤14 alcoholic drinks per week for men), and high consumers (>7 alcoholic drinks per week for women and >14 alcoholic drinks per week for men). Information on mobility limitation was obtained from the EuroQol-5D questionnaire and was defined as having any difficulties with walking in the previous week. Health status was determined by the presence or a history of one or more of the following conditions: cardiovascular disease, COPD, cancer or Parkinson’s disease. Medication use was assessed during a medication interview and was defined as the use of glucose-lowering medication, blood-pressure-lowering medication or lipid-modifying medication. Other covariates included BMI and HbA_1c_, which were obtained from physical examination and laboratory assessment, as described elsewhere [22], and higher intensity physical activity.

### Statistical analysis

Descriptive characteristics of the study sample were summarised as mean with SD or as numbers and percentages. Diabetes duration was described using the median and interquartile range. To examine differences between the groups with NGM, IGM and type 2 diabetes, we conducted *χ*^2^, ANOVA and Kruskal–Wallis tests as appropriate. General linear models were used to obtain adjusted means of the amount of sedentary time, the number of sedentary breaks, the number of prolonged sedentary bouts and the average bout duration, and these were compared between participants with NGM, IGM and type 2 diabetes. Bonferroni-corrected pairwise comparisons were made between the groups. Similar analyses were conducted to compare the adjusted means between participants without metabolic syndrome criteria, those with one or two metabolic syndrome criteria and those with the metabolic syndrome (three or more criteria). To examine associations of the sedentary behaviour variables with glucose metabolism status and the metabolic syndrome, multinomial logistic regression analyses were conducted. Results are reported as ORs with 95% CIs. For both general linear models and multinomial regression analyses an unadjusted model and three adjusted models were fitted. Model 1 was adjusted for sex, age, level of education and waking time. Model 2 was additionally adjusted for smoking status, alcohol consumption, health status and mobility limitation. For glucose metabolism status, model 2 was also adjusted for BMI. Model 3 was additionally adjusted for higher intensity physical activity. For the exposure variables sedentary breaks, prolonged sedentary bouts and sedentary bout duration, model 3 was also adjusted for sedentary time. The exposure variables were checked for normality, which was reasonable. Further, we tested multi-collinearity in our models: no variables had diverged confidence intervals or standard errors, or an unexpected change in the regression coefficient, and the variance-inflating factors were *<* 2.0. All analyses were conducted with IBM SPSS Statistics 22.0 (IBM, Armonk, NY, USA).

## Results

**Fig. 1:**
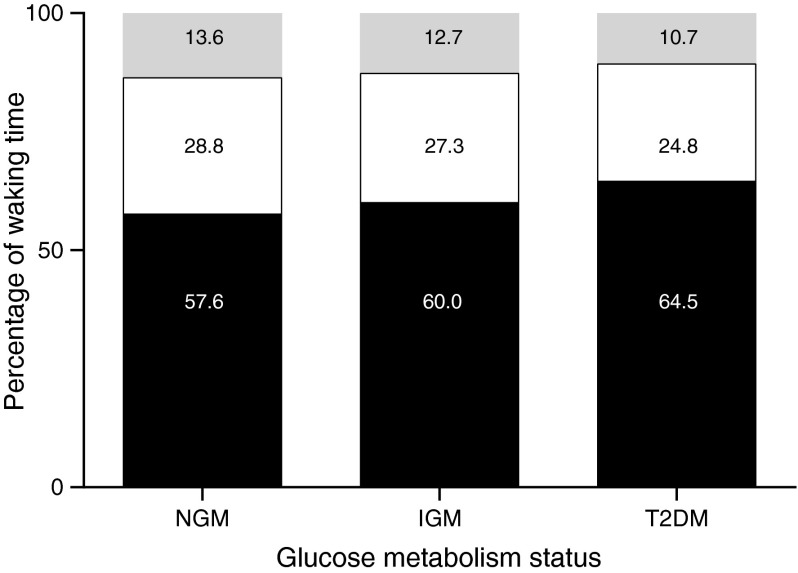
Percentages of waking time spent sedentary, standing and stepping according to glucose metabolism status. Black bars, sitting/lying; white bars, standing; grey bars, stepping. T2DM, type 2 diabetes mellitus

The overall study population consisted of 2,497 participants with an average age of 60.0 ± 8.1 years, 52% of whom were men. Table 1 presents descriptive characteristics in the overall study population and according to glucose metabolism status. A total of 1,395 (55.9%) participants had NGM, 388 (15.5%) had IGM and 714 (28.6%) had type 2 diabetes. Participants with type 2 diabetes were more often current smokers, were less often consumers of high levels of alcohol, had more often a mobility limitation and had a higher BMI compared with participants from the IGM and NGM groups. Those with type 2 diabetes had, on average, an HbA_1c_ of 6.9% (51.9 mmol/mol) and a median duration of diabetes of 6 years (Q1–Q3 = 3.0–12.0). Participants in all groups provided, on average, more than 6 valid days of data with an average waking time of almost 16 h. Figure 1 shows the percentages of waking time spent sedentary, standing and stepping on an average day; these were statistically significantly different between the three groups (*p* < 0.01).

**Table 1:** Descriptive characteristics of the study population

Characteristic	Total *n* = 2,497	NGM *n* = 1,395 (55.9%)	IGM *n* = 388 (15.5%)	T2DM *n* = 714 (28.6%)	*p* value
Sex (% men)	52.0	41.9	54.9	70.2	<0.001
Age (years)	60.0 (8.1)	58.1 (8.1)	61.9 (7.2)	62.7 (7.7)	<0.001
Education level (%)					<0.001
High	38.4	44.9	36.9	26.6	
Medium	28.1	28.5	26.3	28.4	
Low	33.5	26.7	36.9	45.0	
Smoking status (% current smoker)	13.0	12.3	11.3	15.3	<0.001
Alcohol consumption (% high consumer)	25.4	27.5	31.4	17.9	<0.001
Mobility limitation (% limited mobility)	16.5	10.8	16.5	27.6	<0.001
BMI (kg/m^2^)	27.1 (4.5)	25.5 (3.6)	27.7 (4.3)	29.8 (4.9)	<0.001
Health status (% with [history of] cardiovascular disease, COPD, cancer or Parkinsons)	26.8	20.5	26.0	39.4	<0.001
Medication use (%)	52.5	29.4	58.5	94.3	<0.001
Diabetes medication use (%)	n/a	n/a	n/a	79.3	n/a
Diabetes duration (years)^a^ median (Q1–Q3)	n/a	n/a	n/a	6.0 (3.0–12.0)	n/a
HbA_1c_ (%)	5.9 (0.9)	5.4 (0.3)	5.7 (0.4)	6.9 (1.0)	<0.001
HbA_1c_ (mmol/mol)	40.9 (9.8)	35.9 (3.8)	38.6 (4.6)	51.9 (11.1)	<0.001
Metabolic syndrome (%)	39.0	15.8	52.1	77.2	<0.001
Number of valid days (>14 h of monitoring)	6.3 (1.2)	6.4 (1.1)	6.2 (1.1)	6.2 (1.2)	0.001
Waking time (h/day)	15.7 (0.9)	15.7 (0.9)	15.8 (0.8)	15.7 (1.0)	0.183
Higher intensity physical activity (min/day)	22.6 (18.5)	26.8 (19.5)	21.8 (17.3)	14.7 (13.7)	<0.001

Values are means (SD), unless stated otherwise

^a^ *n* = 493 with type 2 diabetes

n/a, not applicable; T2DM, type 2 diabetes mellitus

Table 2 presents the unadjusted and adjusted means of the sedentary behaviour variables according to glucose metabolism status. After adjustment for several confounders, a statistically significant difference of up to 26 min (0.43 h) in sedentary time was seen between the three groups (model 3). The differences in number of sedentary breaks per day were, although statistically significantly in the unadjusted model and model 1, small between the groups in all analyses. The daily number of prolonged sedentary bouts with a duration of 30 min or longer differed significantly between groups in the models up to model 3. After adjustment for sedentary time and higher intensity physical activity in model 3, the number of prolonged sedentary bouts became similar in the three groups. As was seen for the number of bouts, there were statistically significant differences in average sedentary bout duration between the groups in the models up to model 3, but after adjustment for sedentary time and higher intensity physical activity, the duration became comparable between the groups.

**Table 2:** Unadjusted and adjusted means with 95% CI for sedentary behaviour variables according to glucose metabolism status

Variable	NGM (*n* = 1,395)		IGM (*n* = 388)		T2DM (*n* = 714)		*p* value			
	Mean	(95% CI)	Mean	(95% CI)	Mean	(95% CI)	Overall	NGM vs IGM	NGM vs T2DM	IGM vs T2DM
Sedentary time (h/day)										
Unadjusted model	9.06	(9.0, 9.1)	9.46	(9.3, 9.6)	10.10	(10.0, 10.2)	<0.001	<0.001	<0.001	<0.001
Model 1	9.13	(9.0, 9.2)	9.41	(9.3, 9.6)	9.99	(9.9, 10.1)	<0.001	0.005	<0.001	<0.001
Model 2	9.24	(9.2, 9.3)	9.38	(9.2, 9.5)	9.78	(9.7, 9.9)	<0.001	0.334	<0.001	<0.001
Model 3	9.28	(9.2, 9.4)	9.38	(9.2, 9.5)	9.71	(9.6, 9.8)	<0.001	0.761	<0.001	0.001
Sedentary break (no./day)										
Unadjusted model	55.69	(55.0, 56.4)	55.00	(53.5, 56.5)	52.78	(51.7, 53.9)	<0.001	1.000	<0.001	0.042
Model 1	55.24	(54.5, 56.0)	55.22	(53.9, 56.6)	53.54	(52.5, 54.6)	0.029	1.000	0.034	0.153
Model 2	54.43	(53.7, 55.2)	55.51	(54.2, 56.8)	54.98	(53.9, 56.1)	0.377	0.517	1.000	1.000
Model 3	54.42	(53.7, 55.2)	55.53	(54.2, 56.9)	54.97	(53.9, 56.1)	0.363	0.485	1.000	1.000
Prolonged sedentary bout (≥30 min) (no./day)										
Unadjusted model	4.55	(4.5, 4.6)	4.88	(4.7, 5.0)	5.42	(5.3, 5.5)	<0.001	<0.001	<0.001	<0.001
Model 1	4.62	(4.5, 4.7)	4.82	(4.7, 5.0)	5.31	(5.2, 5.4)	<0.001	0.064	<0.001	<0.001
Model 2	4.74	(4.7, 4.8)	4.80	(4.7, 4.9)	5.09	(5.0, 5.2)	<0.001	1.000	<0.001	0.005
Model 3	4.88	(4.8, 4.9)	4.82	(4.7, 4.9)	4.81	(4.7, 4.9)	0.381	1.000	0.599	1.000
Average sedentary bout duration (min)										
Unadjusted model	10.54	(10.4, 10.7)	11.15	(10.8, 11.5)	12.62	(12.3, 12.9)	<0.001	0.008	<0.001	<0.001
Model 1	10.75	(10.6, 10.9)	11.04	(10.7, 11.4)	12.29	(12.0, 12.6)	<0.001	0.412	<0.001	<0.001
Model 2	11.06	(10.9, 11.3)	10.95	(10.6, 11.3)	11.72	(11.4, 12.0)	<0.001	1.000	0.001	0.001
Model 3	11.28	(11.1, 11.4)	10.99	(10.7, 11.3)	11.27	(11.0, 11.5)	0.191	0.256	1.000	0.381

Model 1 adjusted for sex, age, level of education and waking time. Model 2 is additionally adjusted for smoking status, alcohol consumption, health status ([history of] cardiovascular disease, COPD, cancer or Parkinson’s disease), mobility limitation and BMI. Model 3 is additionally adjusted for higher intensity physical activity and for sedentary time for the variables sedentary break, prolonged sedentary bout and average sedentary bout duration

no., number; T2DM, type 2 diabetes mellitus

The unadjusted and adjusted means of the sedentary behaviour variables according to number of metabolic syndrome criteria are presented in Table 3. In all models, a gradual increase in sedentary time was seen when more metabolic syndrome criteria were present. The difference in sedentary time in model 3 was 40 min (0.66 h) per day between participants with no criteria and those with three to five criteria, and this was statistically significantly different even after adjustment for several confounders. In addition, the number of sedentary breaks per day was statistically significantly different in all models, although the difference in model 3 was only two breaks per day. The number of prolonged sedentary bouts did not differ between the groups after adjustment for confounders. The average sedentary bout duration was statistically significantly different between the groups, but the difference was less than 1 min after adjustment for relevant confounders.

**Table 3:** Unadjusted and adjusted means with 95% CIs of sedentary behaviour variables according to number of metabolic syndrome criteria

Variable	0 criteria (*n* = 361)		1–2 criteria (*n* = 1,162)		3–5 criteria (*n* = 974)		*p* values			
	Mean	(95% CI)	Mean	(95% CI)	Mean	(95% CI)	Overall	0 vs 1–2 criteria	0 vs 3–5 criteria	1–2 vs 3–5 criteria
Sedentary time (h/day)										
Unadjusted model	8.69	(8.5, 8.9)	9.18	(9.1, 9.3)	9.96	(9.9, 10.1)	<0.001	<0.001	<0.001	<0.001
Model 1	8.87	(8.7, 9.0)	9.18	(9.1, 9.3)	9.90	(9.8, 10.0)	<0.001	0.003	<0.001	<0.001
Model 2	8.93	(8.8, 9.1)	9.23	(9.1, 9.3)	9.83	(9.7, 9.9)	<0.001	0.003	<0.001	<0.001
Model 3	9.06	(8.9, 9.2)	9.28	(9.2, 9.4)	9.72	(9.6, 9.8)	<0.001	0.047	<0.001	<0.001
Sedentary break (no./day)										
Unadjusted model	57.11	(55.7, 58.5)	55.73	(54.9, 56.5)	52.71	(51.8, 53.6)	<0.001	0.329	<0.001	<0.001
Model 1	56.06	(54.6, 57.5)	55.61	(54.8, 56.4)	53.24	(52.4, 54.1)	<0.001	1.000	0.004	<0.001
Model 2	55.95	(54.5, 57.4)	55.55	(54.8, 56.3)	53.36	(52.5, 54.2)	0.001	1.000	0.011	0.001
Model 3	55.81	(54.4, 57.3)	55.50	(54.7, 56.3)	53.46	(52.6, 54.4)	0.002	1.000	0.030	0.003
Prolonged sedentary bout (≥30 min) (no./day)										
Unadjusted model	4.24	(4.1, 4.4)	4.64	(4.6, 4.7)	5.32	(5.2, 5.4)	<0.001	<0.001	<0.001	<0.001
Model 1	4.42	(4.3, 4.6)	4.64	(4.6, 4.7)	5.26	(5.2, 5.4)	<0.001	0.042	<0.001	<0.001
Model 2	4.47	(4.3, 4.6)	4.68	(4.6, 4.8)	5.20	(5.1, 5.3)	<0.001	0.054	<0.001	<0.001
Model 3	4.85	(4.7, 4.9)	4.83	(4.8, 4.9)	4.88	(4.8, 4.9)	0.438	1.000	1.000	0.597
Average sedentary bout duration (min)										
Unadjusted model	9.80	(9.5, 10.1)	10.68	(10.5, 10.9)	12.42	(12.2, 12.7)	<0.001	<0.001	<0.001	<0.001
Model 1	10.29	(9.9, 10.7)	10.71	(10.5, 10.9)	12.20	(12.0, 12.4)	<0.001	0.138	<0.001	<0.001
Model 2	10.39	(10.0, 10.7)	10.79	(10.6, 11.0)	12.08	(11.9, 12.3)	<0.001	0.160	<0.001	<0.001
Model 3	11.02	(10.7, 11.3)	11.03	(10.9, 11.2)	11.55	(11.4, 11.7)	<0.001	1.000	0.019	<0.001

Model 1 is adjusted for sex, age, level of education and waking time. Model 2 is additionally adjusted for smoking status, alcohol consumption, health status ([history of] cardiovascular disease, COPD, cancer or Parkinson’s disease) and mobility limitation. Model 3 is additionally adjusted for higher intensity physical activity and for sedentary time for the variables sedentary break, prolonged sedentary bout and average sedentary bout duration

no., number

In Table 4 the odds ratios resulting from the multinomial logistic regression analysis for glucose metabolism status and number of metabolic syndrome criteria are shown. Each additional hour of sedentary time was associated with a 1.13 (95% CI 1.05, 1.22) times higher odds for IGM and a 1.46 (1.36, 1.56) times higher odds for type 2 diabetes compared with NGM (model 1). After adjustment for BMI and other covariates in models 2 and 3, the associations remained statistically significant for type 2 diabetes (OR 1.22, 95% CI 1.13, 1.32). The number of sedentary breaks, the number of prolonged sedentary bouts and the average sedentary bout duration were not statistically significantly associated with glucose metabolism status after adjustment for relevant confounders (model 3).

**Table 4:** Associations of sedentary behaviour variables with glucose metabolism status and number of metabolic syndrome criteria

Variable	Unadjusted model		Model 1		Model 2		Model 3	
	OR	(95% CI)	OR	(95% CI)	OR	(95% CI)	OR	(95% CI)
Sedentary time (h)								
Glucose metabolism status								
NGM	ref.		ref.		ref.		ref.	
IGM	1.17	(1.09, 1.25)	1.13	(1.05, 1.22)	1.08	(1.00, 1.17)	1.07	(0.98, 1.16)
T2DM	1.49	(1.41, 1.59)	1.46	(1.36, 1.56)	1.28	(1.18, 1.37)	1.22	(1.13, 1.32)
Metabolic syndrome criteria								
0 criteria	ref.		ref.		ref.		ref.	
1–2 criteria	1.21	(1.12, 1.30)	1.16	(1.07, 1.26)	1.16	(1.07, 1.27)	1.13	(1.04, 1.24)
3–5 criteria	1.63	(1.51, 1.77)	1.59	(1.46, 1.74)	1.53	(1.39, 1.67)	1.39	(1.27, 1.53)
Sedentary breaks (no.)								
Glucose metabolism status								
NGM	ref.		ref.		ref.		ref.	
IGM	1.00	(0.99, 1.01)	1.00	(0.99, 1.01)	1.01	(1.00, 1.02)	1.01	(1.00, 1.02)
T2DM	0.99	(0.98, 0.99)	0.99	(0.98, 1.00)	1.00	(1.00, 1.01)	1.01	(1.00, 1.01)
Metabolic syndrome criteria								
0 criteria	ref.		ref.		ref.		ref.	
1–2 criteria	0.99	(0.99, 1.00)	1.00	(0.99, 1.01)	1.00	(0.99, 1.01)	1.00	(0.99, 1.01)
3–5 criteria	0.98	(0.97, 0.99)	0.98	(0.97, 0.99)	0.99	(0.98, 0.99)	0.99	(0.98, 1.00)
Prolonged sedentary bout (≥30 min) (no.)								
Glucose metabolism status								
NGM	ref.		ref.		ref.		ref.	
IGM	1.16	(1.08, 1.25)	1.10	(1.02, 1.19)	1.04	(0.96, 1.13)	0.94	(0.83, 1.08)
T2DM	1.44	(1.36, 1.53)	1.35	(1.26, 1.45)	1.17	(1.09, 1.26)	0.91	(0.81, 1.03)
Metabolic syndrome criteria								
0 criteria	ref.		ref.		ref.		ref.	
1–2 criteria	1.21	(1.11, 1.31)	1.15	(1.05, 1.25)	1.15	(1.05, 1.25)	1.01	(0.88, 1.17)
3–5 criteria	1.62	(1.48, 1.76)	1.51	(1.38, 1.66)	1.45	(1.32, 1.60)	1.07	(0.91, 1.24)
Average sedentary bout duration (min)								
Glucose metabolism status								
NGM	ref.		ref.		ref.		ref.	
IGM	1.06	(1.02, 1.10)	1.03	(1.00, 1.07)	0.99	(0.96, 1.03)	0.96	(0.92, 1.01)
T2DM	1.17	(1.14, 1.21)	1.13	(1.10, 1.16)	1.05	(1.02, 1.08)	0.99	(0.95, 1.02)
Metabolic syndrome criteria								
0 criteria	ref.		ref.		ref.		ref.	
1–2 criteria	1.11	(1.06, 1.15)	1.07	(1.03, 1.12)	1.07	(1.02, 1.12)	1.03	(0.98, 1.08)
3–5 criteria	1.27	(1.22, 1.32)	1.21	(1.16, 1.27)	1.19	(1.14, 1.25)	1.09	(1.03, 1.15)

Model 1 is adjusted for sex, age, level of education and waking time. Model 2 is additionally adjusted for smoking status, alcohol consumption, health status ([history of] cardiovascular disease, COPD, cancer or Parkinson’s disease) and mobility limitation. Glucose metabolism status is also additionally adjusted for BMI. Model 3 is additionally adjusted for higher intensity physical activity and for sedentary time for the variables sedentary break, prolonged sedentary bout and average sedentary bout duration

no., number; ref, reference; T2DM, type 2 diabetes mellitus

For the number of metabolic syndrome criteria, longer amounts of sedentary time were, after adjustment for several confounders, significantly associated with increased odds of 13% (95% CI 1.04, 1.24) for having one to two criteria, and 39% (1.27, 1.53) for having three to five criteria compared with participants without any criteria. After adjustment for relevant confounders, the analyses of the sedentary behaviour patterns resulted in statistically significant associations of sedentary breaks and average sedentary bout duration with three to five metabolic syndrome criteria in only model 3 (OR_breaks_ 0.99, 95% CI 0.98, 1.00; OR_bout duration_ 1.09, 95% CI 1.03, 1.15). Additional analyses with separate adjustments for higher intensity physical activity and for sedentary time showed that for the number of prolonged sedentary bouts and average sedentary bout duration, the adjustment for sedentary time predominantly caused the change between models 2 and 3.

Additional analyses examining the number of sedentary breaks with a duration of at least 1 min with glucose metabolism status and the metabolic syndrome showed results similar to those for sedentary breaks of any duration; a statistically significant association was seen for three to five metabolic syndrome criteria in model 3 only (OR 0.98, 95% CI 0.96, 1.00) (data not shown).

## Discussion

To our knowledge, our study is the largest in which posture-discriminating accelerometry was used to objectively measure total amount and patterns of sedentary behaviour in a sample of adults comprising participants with type 2 diabetes, IGM or NGM. The results showed that participants with type 2 diabetes had the most sedentary time, up to 26 min more per day compared with participants with IGM or NGM. Each extra hour of sedentary time was associated with increased odds for type 2 diabetes of 22%. No statistically significant differences were observed between participants with NGM and those with IGM. More time spent sedentary was also associated with a 1.13 times higher odds for one to two metabolic syndrome criteria and a 1.39 times higher odds for the metabolic syndrome (three to five criteria), independent of higher intensity physical activity. The number of sedentary breaks per day was highly comparable and not significantly different between the participants with type 2 diabetes, those with IGM and those with NGM. A statistically significant difference in the number of breaks was seen between participants with the metabolic syndrome and those without the metabolic syndrome, but the difference was only two breaks per day. Also, the odds ratio for the metabolic syndrome was statistically significant but small, and therefore not clinically relevant. Similar results were found for average sedentary bout duration: a statistically significant difference in bout duration was seen between participants with the metabolic syndrome and those without the metabolic syndrome, but the difference was less than 1 min. Also, the OR for the metabolic syndrome was statistically significant: a 1.09 times higher odds for three to five criteria when the average sedentary bout was longer. Last, the number of prolonged sedentary bouts did not differ between the groups according to diabetes status or the groups according to the metabolic syndrome, and the odds ratios were not statistically significant.

A major strength of our study was the use of posture-based measurement with the activPAL accelerometer, worn on the thigh, which has been shown to be a highly accurate method for assessing sedentary behaviour [20, 21]. Thus, our estimates of the amounts of sedentary time and the determination of sedentary breaks are more accurate than data based solely on acceleration, which cannot discriminate between postures. Further, we used accelerometry in a large sample of middle-aged and older adults with type 2 diabetes and IGM, which enabled us to examine and quantify associations of several objectively measured sedentary behaviour variables with type 2 diabetes. Also, waterproofed attachment of the activPAL on the thigh enabled us to collect 24 h accelerometry data, which not only resulted in complete data assessments, but could also have improved wear-time compliance, as demonstrated in a recently published study in children [27]. Wear time in our study population was, on average, 6.3 days with 15.7 h of waking time, and 85.3% of our study population provided at least 6 valid days of data. Another strength was adjustment for important confounders including BMI and high-intensity physical activity, which excludes the possibility that these factors account for the associations of sedentary behaviour with type 2 diabetes and the metabolic syndrome. The adjustment for BMI predominantly caused the differences between models 1 and 2, yet BMI could be part of the pathway between sedentary behaviour and type 2 diabetes. Consequently, the analyses could have been subject to overadjustment.

A few limitations should also be mentioned, of which study design is the most important. As our analyses were cross-sectional in nature, causal relationships could not be examined. It may therefore be possible that participants with type 2 diabetes had more sedentary time because of their poorer health. However, when participants with type 2 diabetes on insulin medication (who may be considered to have more severe type 2 diabetes and could for that reason have more sedentary time) were excluded from the analysis, the results did not change. This may suggest that sedentary behaviour at least partly preceded type 2 diabetes, as the associations were similar among participants who did not necessarily have to spend more time sedentary because of their health (data not shown). Furthermore, previous prospective studies have demonstrated that sedentary behaviour predicts markers of insulin resistance [5, 13]. Taken together, these findings support the hypothesis that the direction of the association is predominantly from sedentary behaviour to health outcomes, although large-scale prospective studies are warranted to provide better insights into the directions of the associations. Further, although we adjusted for a broad range of confounding factors, it is possible that some unmeasured factors, for example dietary intake, partly explain the associations. Finally, sedentary behaviour was measured during 1 week only, and this may not truly reflect habitual behaviour.

Several previous studies have used accelerometry to objectively measure sedentary behaviour and examined its associations with the metabolic syndrome. In line with our results, larger amounts of sedentary time have been associated with metabolic risk, although the reported effect sizes were smaller [8, 11, 15–17]. To date, no studies have reported associations between objectively measured sedentary time as exposure variable and type 2 diabetes as outcome measure. However, a meta-analysis of studies with self-reported measures of sedentary behaviour showed a risk of 112% for type 2 diabetes in the group with the highest compared with the lowest amounts of sedentary time [28]. Furthermore, large amounts of objectively measured sedentary time have been associated with (markers of) insulin resistance [5, 6, 10] and, as mentioned earlier, the metabolic syndrome. As these factors are precursors to type 2 diabetes, the results of these studies in combination with the results of the meta-analysis may support our findings of an increased risk for type 2 diabetes with increasing amount of sedentary time. Physiological mechanisms that could explain our findings have not yet been studied extensively, but results from animal studies suggest that responses to contractile (in)activity of muscle cells can play a role in glucose metabolism as reductions in lipoprotein lipase (LPL), an enzyme that contributes to the metabolism and transport of lipids, were seen after periods of inactivity [29, 30].

Few observational studies have reported associations between patterns of sedentary behaviour and metabolic health, but the findings that have been reported are inconsistent. Some studies have shown associations between the number of sedentary breaks and metabolic risk factors [6, 15, 18], while others have not [5, 16]. These inconsistencies could be caused by different methods for measuring breaks, which can be based on change in acceleration or on change in posture. Also, differences exist in the definition of a break, which can be any interruption of sedentary time or interruptions of at least 1 min, although we did not find different results for sedentary breaks of any duration and breaks of at least 1 min. Further, comparability of studies is hampered by differences in adjustment strategies and, furthermore, associations could be different among younger and older adults and among adults with and without (a higher risk for) type 2 diabetes, because their metabolic profiles differ. Studies on prolonged sedentary bouts and sedentary bout duration are scarce and no study on associations with type 2 diabetes has been reported. However, the studies of Bankoski [15] and Healy [7] show no statistically significant associations of bout length or number of bouts ≥30 min with metabolic variables, except waist circumference. Further, in our study we used three measures for expressing the sedentary behaviour pattern, but other measures could also be used to study the pattern of sedentary time [31]. In order to compare studies examining associations of numbers of both sedentary breaks and bouts, future studies should use similar measures, and ideally adjust for similar confounders.

To conclude, this was the largest study that objectively measured total amount and patterns of sedentary behaviour in a sample of adults with type 2 diabetes, IGM or NGM. The results showed that an extra hour of sedentary time was associated with increased odds of 22% for type 2 diabetes and of 39% for the metabolic syndrome, independent of high-intensity physical activity. The pattern in which sedentary time was accumulated, as expressed by number of sedentary breaks, number of prolonged sedentary bouts and average sedentary bout duration, was only weakly associated with an increased risk for the metabolic syndrome. Future studies in participants with type 2 diabetes should be conducted to confirm our results, and to explore dose–response relationships and causality. Nevertheless, our findings could have important implications for public health as they suggest that sedentary behaviour may play a significant role in the development and prevention of type 2 diabetes, independent of high-intensity physical activity. Consideration should be given to including strategies to reduce the amount of sedentary time in diabetes prevention programmes.

## Abbreviations

COPD: Chronic obstructive pulmonary disease
IGM: Impaired glucose metabolism
MET: Metabolic equivalent
NGM: Normal glucose metabolism
